# Community-Based Screening for Infantile Anemia in an Okinawan Village, Japan

**DOI:** 10.1155/2011/278371

**Published:** 2010-11-30

**Authors:** Tomiko Hokama, Chiemi Yogi, Colin W. Binns, Andy H. Lee

**Affiliations:** ^1^Graduate School of Health Sciences, Faculty of Medicine, University of the Ryukyus, 207 Uehara, Nishihara, Okinawa 903-0215, Japan; ^2^176 Aza Toma, Nakagusuku Village, Okinawa 901-2416, Japan; ^3^School of Public Health, Curtin Health Innovation Research Institute, Curtin University, GPO Box U 1987, Perth, WA 6845, Australia

## Abstract

Infancy is a vulnerable age group for anemia throughout the world. However, community-based screening for infantile anemia is seldom reported. This study determined the prevalence of anemia among infants in an Okinawan village from 2003 to 2008, in relation to secondary prevention of the condition. The prevalence among infants aged 3–5, 6–12 and 16–23 months was 12.3%, 15.8%, and 4.2%, respectively, based on cross-sectional surveys (*n* = 3070), and was 11.0%, 17.2%, and 3.9% according to another retrospective cohort study (*n* = 511). The relatively low prevalence of anemia at early childhood suggested that previous detection and treatment through early and late infantile screening had been successful.

## 1. Introduction

Infancy and early childhood are vulnerable age groups for iron deficiency anemia throughout the world. Anemia, with and without iron deficiency, affects child development and is a potentially preventable condition. International agencies continue to emphasize the importance of programs to address anemia in mothers and children [1]. It is estimated that anemia may affect 40–50% of children worldwide [2]. The risk factors of anemia include low birth weight, low income family, and minority ethnic group [3]. It is also important to diagnose and manage iron deficiency because of its effects on cognitive development and increased morbidity in children [4, 5]. The American Academy of Pediatrics recommends screening for all infants between the ages of 9–12 months and then 6 months later whereas for children at high risk, screening is needed once a year from age 2 to 5 years. The Centers for Disease Control and Prevention and the American Academy of Family Physicians also suggested selective anemia screening for preterm and low birth weight infants [6]. 

Okinawa is economically behind other Japanese prefectures. According to a report from the Okinawa prefecture government office, the per capita income of this island prefecture (2.02 million yen) is approximately 70% of that of Japan (2.87 million yen) in 2005. Its prevalence of low birth weight infants (10.9%) is also higher than Japan overall (9.5%), though these rates have been increasing [7]. Community-based screening of anemia has been conducted in Okinawa since 1973. The purpose of this study is to determine the most recent prevalence of infantile anemia in an Okinawan village. The findings are useful in relation to secondary prevention of the condition.

## 2. Materials and Methods

Information on the anemia screening program was obtained from existing health records of Nakagusuku, which is a typical village located at the central part of the main island of Okinawa. It has a population of 17,000 with an annual birth of about 200 infants. Cross-sectional surveys were conducted of infants who received anemia screening test when they attended their regular health examinations provided at community health and welfare centers as a free child care service by the municipal. These examinations were undertaken in early infancy (3 to 5 months, *n* = 942), late infancy (6 to 12 months, *n* = 898), and early childhood (16 to 23 months, *n* = 980) during 2003–2008. Another retrospective cohort study was undertaken on 511 infants who were screened for anemia at these three occasions during the same period. Length and weight were measured using a standard anthropometric protocol, while capillary blood hemoglobin (Hb) concentration was determined by the cyanmetho-hemoglobin method.

Anemia of infants was defined by an Hb concentration being less than 110 g/L. Infants with Hb < 110 g/L were provided with dietary counseling by a qualified dietitian whereas those having an Hb reading less than 100 g/L were referred to a pediatrician for further examination and treatment. Statistical analysis was performed using the SPSS package version 17.

## 3. Results

**Figure 1:**
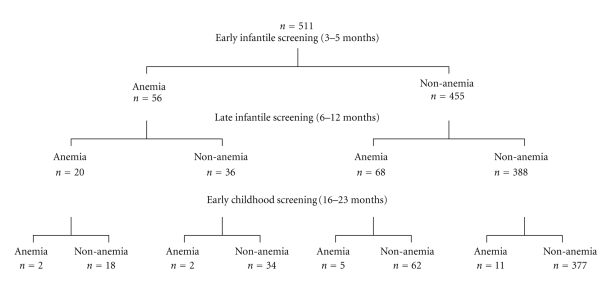
Flowchart of the anemia screening program in the Okinawan village for the cohort study.

Table 1 presents the Hb concentration and other characteristics of the subjects, including the feeding method at the time of examination. Based on the cross sectional surveys, the prevalence estimates of anemia in early infancy, late infancy, and early childhood were 12.3% (116 out of 942), 15.8% (142 out of 898), and 4.2% (41 out of 980), respectively. According to the retrospective cohort survey, the rates in early infancy, late infancy, and early childhood were 11.0% (56 out of 511), 17.2% (88 out of 511), and 3.9% (20 out of 511), respectively.  Figure 1 shows the flowchart of the anemia screening program in Nakagusuku for the retrospective cohort study. The late infant screening result confirmed that anemia had been improved in 36 infants (64.3%) among the 56 anemic infants detected at early infant screening while the early childhood screening had led to improvement in 80 infants (91%) among the 88 anemic infants detected at late infant screening.

## 4. Discussion

A previous screening study of 356 infants in the Nakagusuku Village was undertaken in the 1990s; the prevalence of anemia was 22.5% in late infancy and 11% in early childhood [8]. In 2002-2003, the rate in the same village (*n* = 203) decreased to 16.8% and 7.5%, respectively [9]. The prevalence in Okinawa was still high when compared to Tokyo [10]. Nevertheless, current screening results in the same Okinawan village indicated that the prevalence of anemia in late infancy and early childhood had decreased. This improvement might be due to the continuation of screening program that has been in placed since the 1970s and the improving economic condition in Okinawa.

The decrease in anemia over time suggested that community-based screening could play a role in the prevention of late infantile anemia. Anemia remains a problem in Okinawa, so it should be regarded as a pediatric priority. It is important to maintain breast milk supply as well as appropriate complementary food to prevent late infantile anemia. The purpose of the screening for anemia is to detect and treat anemic infants and then prevent later recurrence. The cohort study showed that 36 (64.3%) infants who were screened and treated for anemia at the early infancy screening recovered after treatment. However, another 20 (35.7%) infants were still anemic at late infancy screening, suggesting that further intervention was necessary. By the time of young childhood screening, most (91%) of the anemia cases had been successfully treated with diet counseling and iron preparations for iron deficiency cases. In conclusion, the screening program for infantile anemia should be continued in Okinawa in conjunction with nutritional guidance to mothers for the prevention of childhood anemia.

## Figures and Tables

**Table 1:** Characteristics of infants in the Okinawan village, Japan.

Cross sectional survey	3–5 months	6–12 months	16–23 months
*n*	942	898	980
Male (%)	471 (50%)	443 (49.3%)	478 (48.8%)
Mean birth weight ± SD g	3026 ± 425	2993 ± 438	2999 ± 442
Mean gestational age ± SD week	38.9 ± 1.7	38.8 ± 1.8	38.8 ± 1.7
Mean maternal age ± SD years	29.7 ± 5.4	30.3 ± 5.4	31.2 ± 3.5
Mean Hb ± SD g/L	121 ± 11	120 ± 11	124 ± 9
Birth order (%)			
1	441 (46.8%)	431 (48.0%)	442 (45.1%)
2	284 (30.1%)	271 (30.2%)	284 (29.0%)
3	217 (23.1%)	196 (21.8%)	254 (25.9%)
Feeding method at screening (%)			
Breast-fed	237 (25.2%)	151 (16.8%)	
Mixed-fed	334 (35.5%)	158 (17.6%)	
Formula-fed	371 (39.4%)	589 (65.5%)	

Cohort study			

*n*	511		
Male (%)	237 (46.4%)		
Mean birth weight ± SD g	3023 ± 429		
Mean gestational age ± SD week	38.8 ± 1.7		
Mean maternal age ± SD years	30.1 ± 5.5		
Mean Hb ± SD g/L	121 ± 10	120 ± 11	125 ± 9
Birth order (%)			
1	252 (49.3%)		
2	142 (30.1%)		
3	117 (22.9%)		
Feeding method at screening (%)			
Breast-fed	109 (21.3%)	79 (15.5%)	
Mixed-fed	210 (41.1%)	80 (15.7%)	
Formula-fed	192 (37.6%)	352 (68.9%)	
